# Supplementary material for: Emergence of periodic circumferential actin cables from the anisotropic fusion of actin nanoclusters during tubulogenesis
Source: Nat Commun. 2024 Jan 24;15:464. doi: 10.1038/s41467-023-44684-z (PMC10808230; doi:10.1038/s41467-023-44684-z)
Supplement: Supplementary file 1 — Supplementary Information [file 41467_2023_44684_MOESM1_ESM.pdf]

Supplementary Information for

Emergence of periodic circumferential actin cables from the anisotropic fusion of actin nanoclusters during tubulogenesis

Sayaka Sekine[1,2,†], Mitsusuke Tarama[3,4,†], Housei Wada[1], Mustafa M Sami[1,5], Tatsuo Shibata[3], and Shigeo Hayashi[1,6]

1, Laboratory for Morphogenetic Signaling, RIKEN Center for Biosystems Dynamics Research, Kobe, Japan.
2, Laboratory for Histogenetic Dynamics, Graduate School of Life Sciences, Tohoku University, Sendai, Japan.
3, Laboratory for Physical Biology, RIKEN Center for Biosystems Dynamics Research, Kobe, Japan.
4, Department of Physics, Faculty of Science, Kyusyu University, Fukuoka, Japan.
5, Physics and Biology Unit, Okinawa Institute of Science and Technology Graduate University, Okinawa, Japan.
6, Kobe University Graduate School of Science, Kobe, Japan.
[†]These authors contributed equally: Sayaka Sekine, Mitsusuke Tarama.
*Correspondence should be addressed to S.S. (sayaka.sekine.d7@tohoku.ac.jp) and M.T. (tarama.mitsusuke@phys.kyushu-u.ac.jp).

This PDF file includes:

Supplementary Figures 1-7
Supplementary Tables 1-6
Supplementary Notes for the theoretical model
Supplementary References

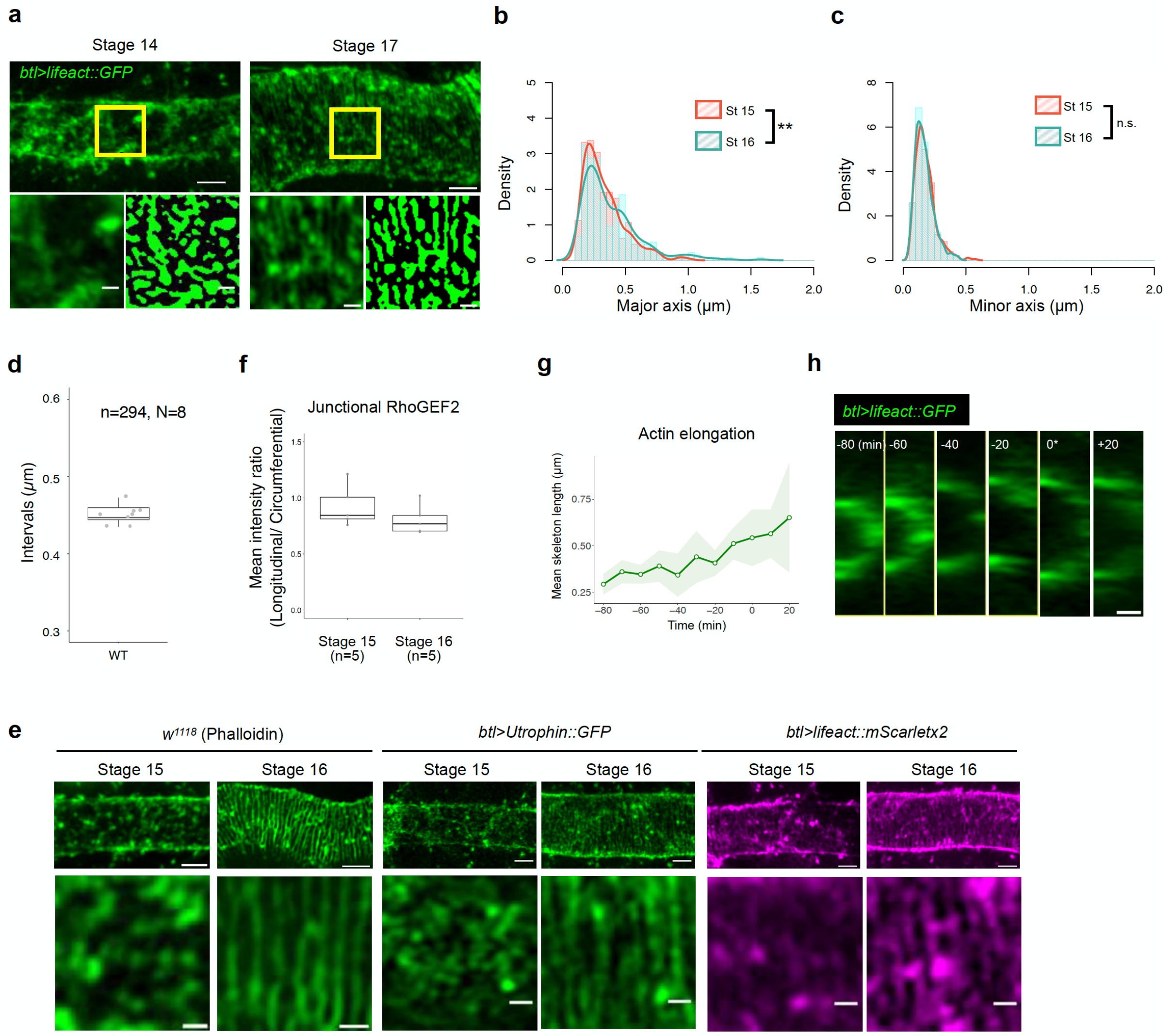

Supplementary Figure 1: Cluster-to-cable transition of cortical actin pattern in the expanding tracheal tube. (a) The actin pattern visualized by lifeact::GFP in the fixed embryos at stages 14 and 17, respectively. The lower square images indicate a magnified view of the yellow square and its binarized image. Three experiments were repeated independently. Scale bars: 2 μm (upper) and 0.5 μm (lower). (**b, c**) Comparison of the cluster-fitted ellipses between stage 15 (n=356, N=22) and stage 16 (n=271, N=21) with length of the major axis (**b**) and length of the minor axis (**c**). For statistical analyses, two-sided two-sample Kolmogorov-Smirnov test was performed. p=0.00981 (**b**) and p=0.329 (**c**). **$p < 0.01$. See Methods' *Cluster analysis* for detail. (**d**) Average interval of the periodic circumferential cables at early stage 16. Boxplots represent median plus minima and maxima with lower and upper quantiles. The number of cables (n) and embryos (N) counted for the measurement are indicated in the plot. (**e**) The actin pattern visualized by Phalloidin555, Utrophin::GFP, and lifeact::mScarletx2 in the fixed embryos at stages 15 and 16, respectively. The lower square images indicate magnified views. Scale bars: 2 μm (upper) and 0.5 μm (lower). (**f**) The mean intensities of RFP::RhoGEF2 at vicinity of longitudinal junctions or circumferential junctions are measured and those ratio are plotted. The accumulation of RhoGEF2 to the longitudinal junction that is seen at larval stage was not observed at embryonic stages 15 nor 16. Boxplots represent median plus minima and maxima with lower and upper

quantiles. (**g**) The transition of average skeleton length (green line) and S.D. (light grey lines) are indicated. n=6 (-80, -70, -60, -50, -40 min), 7 (-30 min), 9 (-20, -10, 0 min), 8 (10 min), or 5 (20 min) biologically independent samples. (**h**) The transverse (yz) images of the tracheal tube during expansion period, stage 15. The time point of circumferential cables formed was defined as 0 min, and the relative time points are written above. Scale bar, 1 µm.

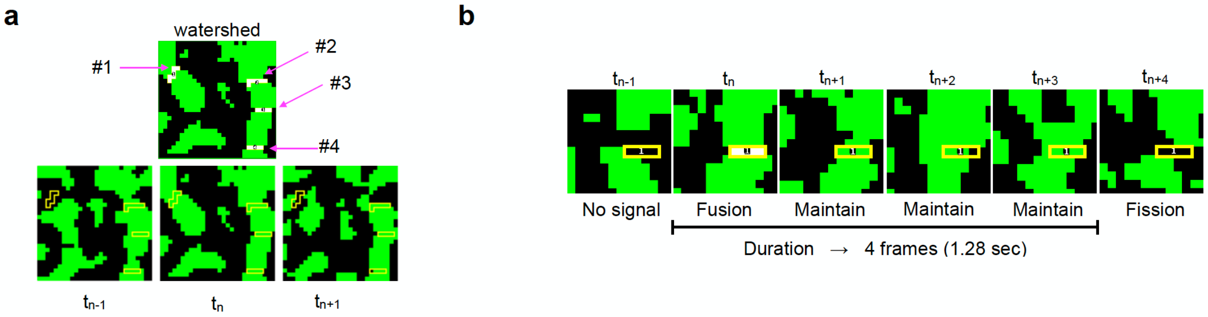

Supplementary Figure 2: Detection of the nanocluster fusion sites and measurement of the duration. (**a**) The potential fusion sites of the actin nanoclusters at time point t_n are selected by watershed segmentation (#1 - 4). Then the actin signals of a single frame before (t_{n-1}) at the potential fusion sites were checked. If the actin signal did not exist at t_{n-1}, the boundary is defined as a newly formed "fusion site" (#1). (**b**) The fusion site was tracked for the subsequent 11 frames to count a number of frames that actin signals were maintained. In this case, the duration is 4 frames (1.28 sec).

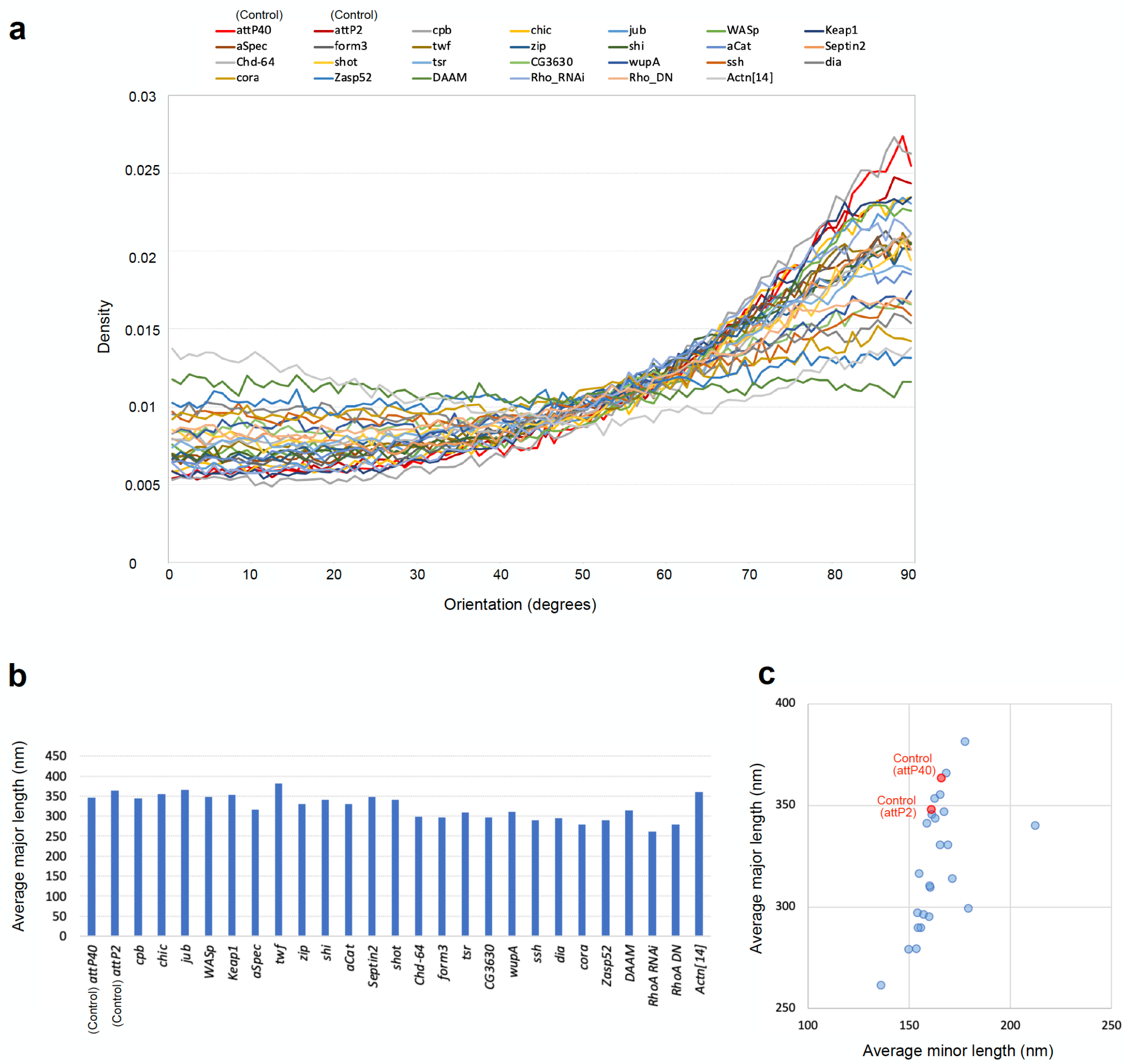

Supplementary Figure 3: The quantification of patterning defect mutants isolated from RNAi screen. (**a**) The distribution of orientation of the actin patterns quantified by OrientationJ in RNAi-expressing tracheal cells at early stage 16. The attP40 (red) and attP2 (dark red) are controls. The target genes of RNAi are written above except Rho_DN: dominant negative form of RhoA (*RhoA*^{N19}); Actn[14]: mutant allele of the *α-actinin*. Each line indicates an average values of multiple ROIs (number of ROIs is listed in Supplementary Table 2). (**b**) Average major length measured by particle analysis of control or genetically manipulated tracheal cells at early stage 16. (**c**) The actin patterns are plotted with two parameters: the average minor length and the average major length. Each circle indicates different genetic conditions: control (red), RNAi lines or other genetic manipulation (light blue). Source data are provided as a Source Data file.

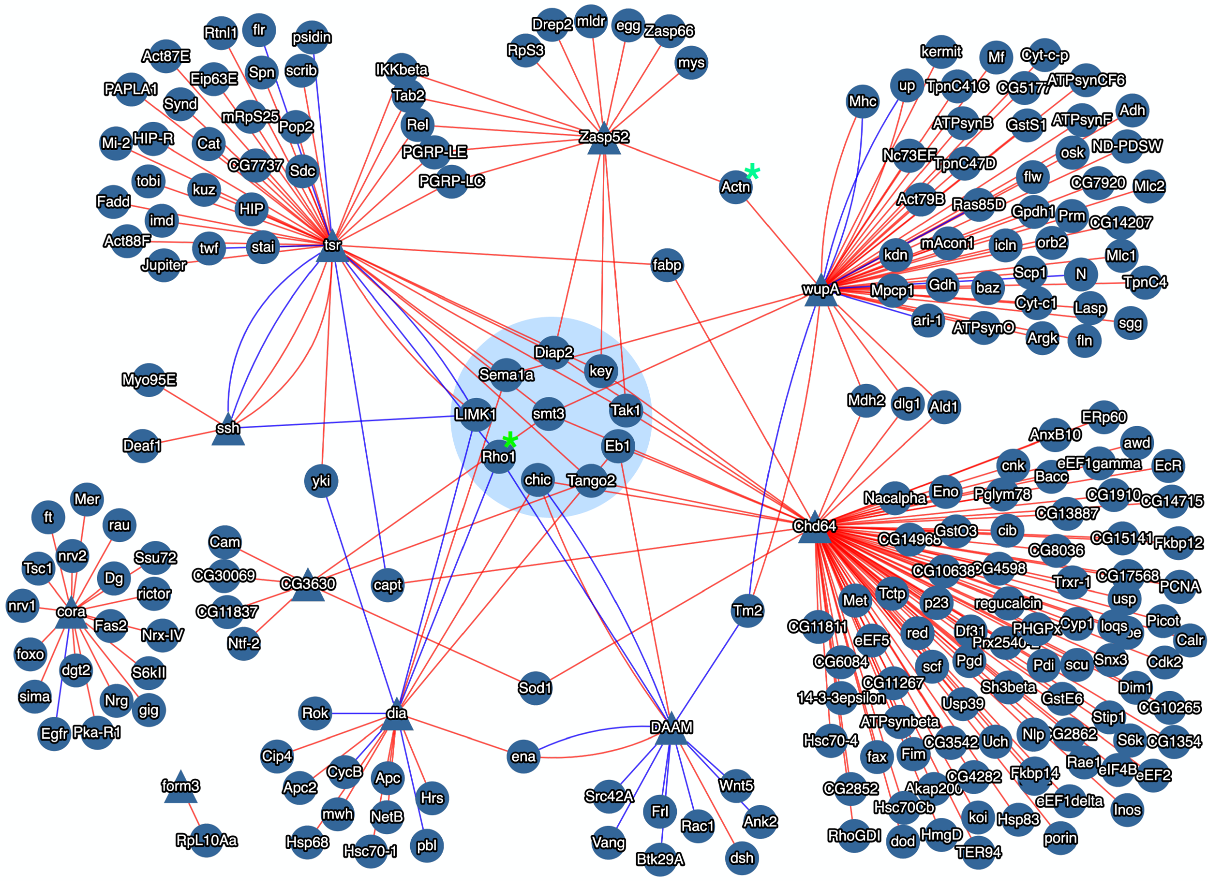

MIST ver 5.0 (April 2020) https://fgrtools.hms.harvard.edu/MIST/

Input genes (triangle, screen positives):
zasp52, wupA, Chd64, DAAM, dia, CG3630, ssh, tsr, cora, form3 (10 genes)

Filtering:
PROTEIN-PROTEIN interactions: Filter OUT LOW rank results
PROTEIN-PROTEIN interactions Interlogs: protein-protein interactions from other species: filter out low and moderate
Genetic interactions: Filter out low
Genetic interactions Interlogs: genetic interactions from other species: filter out low and moderate

Genes that connected more than 3 genes (inside a blue circle):
smt3, Tak1, key, Diap2, sema1a, Eb1, Rho1, LIMK1, Tango2

Supplementary Figure 4: An interaction map of the screen-positive and related genes based on physical and genetic interactions database. Physical interaction (blue line) and genetic interaction (red line) linked 8 out of 10 screen-positive proteins. The downregulation of Rho1 (RhoA, green asterisk) or knockout for actn (green asterisk) also caused circumferential cable defects.

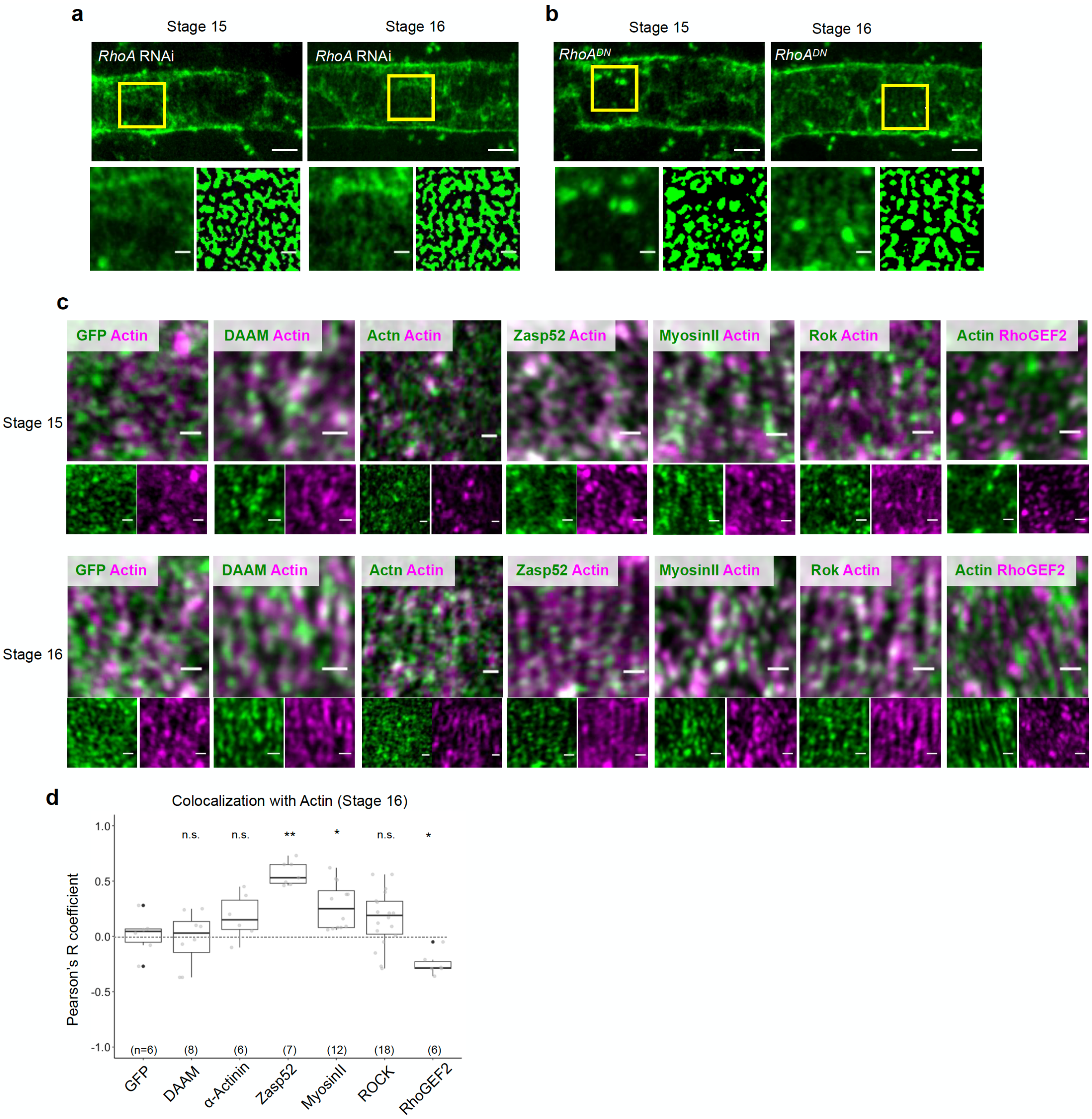

Supplementary Figure 5: The colocalization of screen-positive proteins and actin in the apical cortex. (**a, b**) Confocal projection images and binarized images of RNAi or dominant-negative form of RhoA-expressing tracheal cells, respectively. Scale bars: 2 µm (upper) and 0.5 µm (lower). (**c**) The colocalization of actin and fluorescent protein-tagged proteins at stages 15 and 16. The actin is labelled by lifeact::mScarletx2 except the case with RFP::RhoGEF2, in which used lifeact::GFP. Scale bar, 0.5 µm. (**d**) The colocalization between the proteins with actin at stage 16 were evaluated by Pearson's correlation coefficient. n indicates a biologically independent sample. Boxplots represent median plus minima and maxima with lower and upper quantiles. P values were determined using two-sided Student's t-test. p=0.764 (DAAM), p=0.176 (α-Actinin), p=1.99e-4 (Zasp52), p=0.0195 (Myosin II), p=0.150 (ROCK) or p=0.0641 (RhoGEF2). *$p < 0.05$, **$p < 0.01$. Source data are provided as a Source Data file.

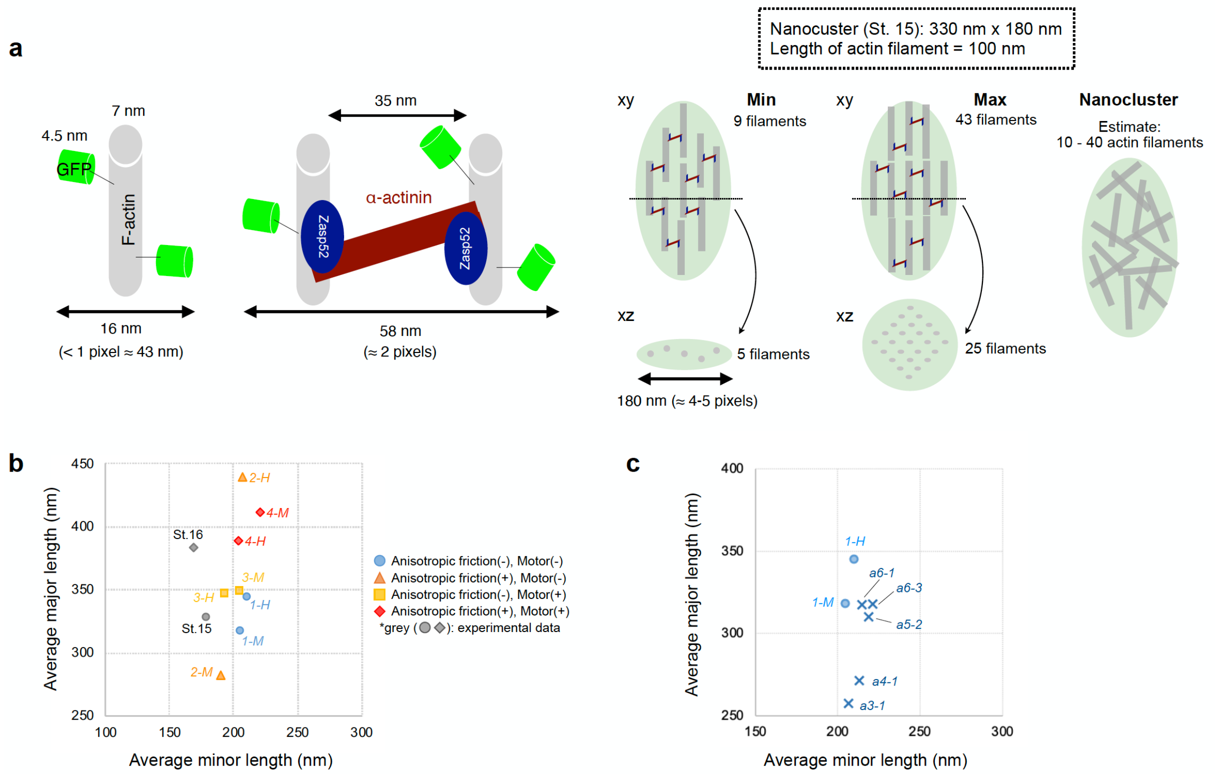

Supplementary Figure 6: The coarse-grained molecular dynamics simulation demonstrated self-assembly of clusters. (a) Schematics of a lifeact::GFP-labeled actin filament and two actin filaments cross-linked by α-Actinin and Zasp52. Using the molecular size reported in literatures, the complex of the two crosslinked actin filaments is estimated as 58 nm in width, which is larger than 1 pixel length (≈ 43 nm), and thus, should be detected by 2 pixels in the image. Then, the cross section (xz) of the actin nanocluster (180 nm) should contain 5 to 25 filaments that align perfectly. Assuming that the actin filament length is 100 nm, a single nanocluster should contain 10 to 40 filaments on average. (b, c) The average minor and major length of actin clusters. Each point corresponds to the simulation in Fig. 4b or Supplementary Fig. 7a. The denoted simulation conditions are also shown in Supplementary Table 5. For comparison, the results of the experiment at stages 15 and 16 are included in b. Source data are provided in Supplementary Table 3.

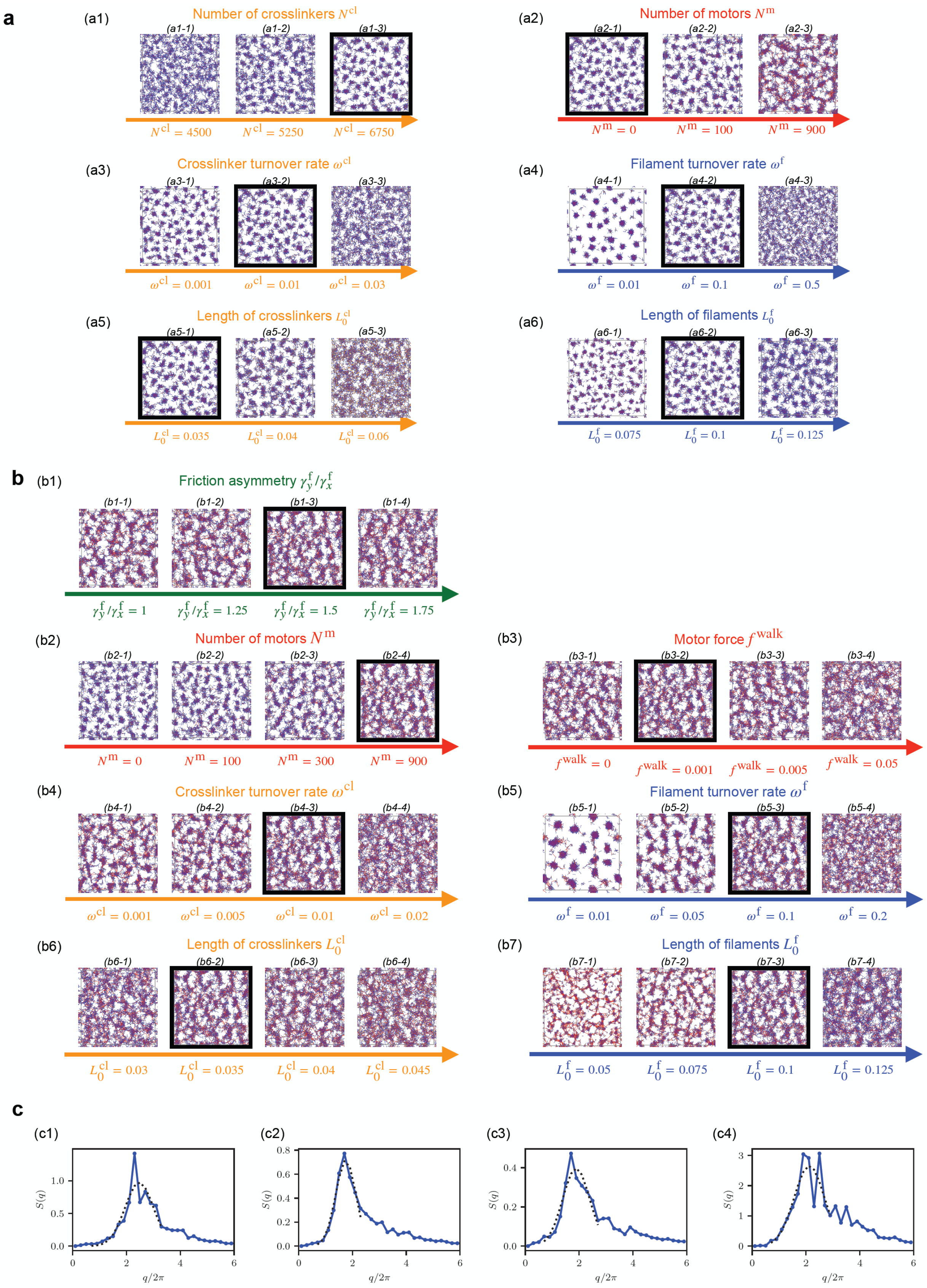

Supplementary Figure 7: The agent-based model simulation reproduced nanocluster-to-cable transition. (a) The simulation results showing how the actin nanoclusters are self-organized in the

absence of motors (a1, a3-a6), and the impact of motors on the cluster formation (a2). The snapshots with black frame are same as the snapshot (1-M) in Fig. 4b in the main text. (b) The simulation results concerning actin stripe formation. The role of the friction asymmetry (b1) and other parameters under the existence of the friction asymmetry (b1-b7) are visualized. The snapshots with black frame are same as the snapshot (4-M) in Fig. 4b in the main text. (c) The interval of the structure estimated by fitting the structure factor S(q) by the gaussian function around the peak. (c1) The clusters, (c2) the labyrinth pattern, (c3) the stripe pattern, and (c4) the clusters for a low filament turnover rate ($\omega^f = 0.01$), corresponding to the snapshots (1-M), (3-M), and (4-M) in Fig. 4b and (a4-1) in Supplementary Fig. 7a, respectively.

Supplementary Table 1. Quantification of actin pattern in the fixed embryos at Stages 15 and 16 (Figure 1 e-g, Figure 4c, Supplementary Figure 6a-c)

		Number	OrientationJ	Cluster analysis (ellipse-fitting)				
		of ROI	Median Orientation	Number of cluster	Area (μm2)	Major length (μm)	Minor length (μm)	Aspect Ratio
Stage 15	Mean	22	44.5°	356	0.0530	0.3286	0.1780	1.9458
	(SD)				0.0514	0.1615	0.0795	0.8754
Stage 16	Mean	21	66.5°	271	0.0576	0.3835	0.1682	2.4241
	(SD)				0.0564	0.2309	0.0717	1.4841

Supplementary Table 2. Quantitative results of the actin pattern of RNAi lines (Figure 3a, Supplementary Figure 3a-c)

(Light blue: RNAi lines with strong actin cable formation defect (screen positives), LL: Larval lethal)

gene	Lethality**	Number	OrientationJ	Cluster analysis (ellipse-fitting)			
		of ROI	Median Orientation	Number of cluster	Average major length (nm)	Average minor length (nm)	Aspect ratio
attP40 (control)	viable	18	64.5	289	345.57	161.02	2.478
attP2 (control)	viable	24	63.5	405	363.37	166	2.458
cpb	semi-LL	24	66.5	418	343.64	162.86	2.499
chic	LL	18	63.5	310	355.31	165.26	2.43
jub	LL	18	62.5	314	366.05	168.12	2.432
WASp	LL	24	61.5	432	347.77	160.87	2.41
Keap1	LL	30	63.5	526	353.54	162.56	2.534
aSpec	LL	21	60.5	408	316.47	154.77	2.297
twf	LL	18	59.5	321	381.54	177.56	2.391
zip	LL	18	59.5	299	330.58	169.1	2.252
shi	LL	18	59.5	358	341.13	158.56	2.381
aCat	LL	24	58.5	447	330.64	165.28	2.255
Septin2	LL	18	57.5	334	347.02	167.22	2.321
shot	LL	18	57.5	264	340.26	212.23	1.981
Chd-64	LL	24	57.5	393	299.28	179.12	2.107
form3	LL	24	60.5	469	297.19	153.95	2.153
tsr	LL	24	57.5	460	309.54	160.42	2.186
CG3630	LL	30	54.5	633	296.45	156.93	2.077
wupA	LL	18	54.5	378	310.36	160.15	2.247
ssh	LL	18	52.5	304	289.87	155.78	2.001
dia	LL	18	51.5	365	295.37	159.62	2.101
cora	LL	24	50.5	497	279.42	153.48	2.018
Zasp52	LL	18	48.5	432	289.91	154.24	2.081
DAAM	LL	18	44.5	298	314	171.33	1.988
RhoA RNAi	n.d.	18	62.5	364	261.63	136.07	2.117
RhoA [DN]	n.d.	24	54.5	531	279.32	149.51	2.055
actn[14]	viable	21	42.5	407	359.84	176.25	2.323
Myosin II[DN]	n.d.	24	54.4	497	325.64	171.16	2.034

Supplementary Table 3. Conditions of simulations and the results of image analysis (Figure 4 b, c, Supplementary Figures 6 b, c, 7a)

*D: dispersed, N: nanocluster, F: fused, NF: nanocluster & fused

**Cluster analysis was not applied if the pattern was disperse (D)

	Conditions							Results					
									OrientationJ			Cluster analysis (ellipse-fitting) **	
Corresponding figures	#crosslinker	Friction	#motor	Filament turnover	Crosslinker turnover	Filament length (μm)	Crosslinker length (μm)	Pattern*	Median orientation (°)	Dominant direction (°)	Coherency	Average minor length (nm)	Average major length (nm)
Fig4b-1-L	4500	1	0	0.1	0.01	0.1	0.0175	D	41	17.4	0.08	n.d.	n.d.
Fig4b-1-M	6750	1	0	0.1	0.01	0.1	0.0175	N	42	24.2	0.05	204.5	318.5
Fig4b-1-H	7500	1	0	0.1	0.01	0.1	0.0175	N	39	2.3	0.07	209.7	345.1
Supplementary Fig. 7a4-1	6750	1	0	0.01	0.01	0.1	0.0175	N	44	39.8	0.02	213.1	271.4
Supplementary Fig. 7a4-3	6750	1	0	0.5	0.01	0.1	0.0175	D	49	74.2	0.09	n.d.	n.d.
Supplementary Fig. 7a3-1	6750	1	0	0.1	0.001	0.1	0.0175	N	43	55.9	0.03	206.4	257.6
Supplementary Fig. 7a3-3	6750	1	0	0.1	0.3	0.1	0.0175	D	42	6.0	0.06	n.d.	n.d.
Supplementary Fig. 7a6-1	6750	1	0	0.1	0.01	0.075	0.0175	N	37	12.1	0.04	214.9	317.4
Supplementary Fig. 7a6-3	6750	1	0	0.1	0.01	0.125	0.0175	N	45	37.0	0.02	221.3	317.9
Supplementary Fig. 7a5-2	6750	1	0	0.1	0.01	0.1	0.02	N	49	81.7	0.02	218.8	310.1
Supplementary Fig. 7a5-3	6750	1	0	0.1	0.01	0.1	0.03	D	50	71.7	0.06	n.d.	n.d.
Fig4b-2-L	4500	1.5	0	0.1	0.01	0.1	0.0175	D	45	57.9	0.04	n.d.	n.d.
Fig4b-2-M	6750	1.5	0	0.1	0.01	0.1	0.0175	N	54	71.6	0.05	190.3	282.9
Fig4b-2-H	7500	1.5	0	0.1	0.01	0.1	0.0175	F	59	85.3	0.15	206.9	439.6
Fig4b-3-L	4500	1	900	0.1	0.01	0.1	0.0175	D	45	62.4	0.05	n.d.	n.d.
Fig4b-3-M	6750	1	900	0.1	0.01	0.1	0.0175	NF	59	84.9	0.17	205.3	349.5
Fig4b-3-H	7500	1	900	0.1	0.01	0.1	0.0175	NF	39	20.6	0.12	192.7	346.6
Fig4b-4-L	4500	1.5	900	0.1	0.01	0.1	0.0175	D	52	62.4	0.15	n.d.	n.d.
Fig4b-4-M	6750	1.5	900	0.1	0.01	0.1	0.0175	F	67	84.8	0.30	221.2	411.4
Fig4b-4-H	7500	1.5	900	0.1	0.01	0.1	0.0175	F	57	82.8	0.13	204.3	389.3
St15	–	–	–	–	–	–	–	N	44.5	n.d.	n.d.	178.0	328.6
St16	–	–	–	–	–	–	–	F	66.5	n.d.	n.d.	168.2	383.5

Supplementary Table 4. Results of structure intervals estimated from the structure factor (Supplementary Figure 7c)

	A	μ/2π	σ	interval (nm)
Supplementary Fig. 7c1 (1-M)	1.28851752	2.4760336	0.52752849	404
Supplementary Fig. 7c2 (3-M)	0.6686629	1.75146809	0.37248651	571
Supplementary Fig. 7c3 (4-M)	0.49937622	1.89140416	0.5010602	529
Supplementary Fig. 7c4 (a4-1)	3.73424732	2.12315061	0.56089287	471

Supplementary Table 5. Results of motion analysis of small clusters (Figure 4d, Figure 5a, d)

	|Δx| (nm)	|Δy| (nm)	|Δy|/|Δx|	distance (nm)	n (N)
Simulation					
Fig4d-1-M	18.2 (± 25.3)	17.6 (± 23.1)	0.97	28.4 (± 31.8)	1461 (1)
Fig4d-2-M	19.4 (± 26.5)	21.8 (± 32.7)	1.13	32.8 (± 39.4)	1134 (1)
Fig4d-4-M	18.4 (± 18.1)	26.6 (± 32.9)	1.45	35.9 (± 34.1)	152 (1)
Stage 15 (wild-type embryo)					
-40 min	75.8 (± 70.3)	81.9 (± 77.0)	1.08	124.1 (± 88.9)	314 (4)
-30 min	63.7 (± 58.4)	86.8 (± 82.6)	1.36	118.1 (± 88.7)	372 (4)
-20 min	63.4 (± 62.0)	83.0 (± 79.6)	1.31	116.1 (± 87.2)	333 (3)
-10 min	73.3 (± 70.0)	102.2 (± 94.1)	1.39	141.3 (± 97.9)	317 (4)
0 min	62.6 (± 65.9)	107.4 (± 104.0)	1.72	137.0 (± 108.7)	322 (4)
Stage 16 (RNAi-expressing embryo)					
Control	49.3 (± 52.8)	78.8 (± 72.6)	1.6	103.1 (± 77.8)	352 (6)
zasp52 RNAi	82.3 (± 79.0)	80.4 (± 71.2)	0.98	129.2 (± 88.8)	977 (10)
DAAM RNAi	60.9 (68.1)	58.4 (72.2)	0.96	94.7 (89.5)	463 (6)
Myosin II[DN]	79.9 (± 71.6)	95.6 (± 80.9)	1.2	139.2 (± 88.4)	955 (13)

Supplementary Table 6. Reagents and genotypes of fly strains used in this study.

Reagents	Source/ Identifiers/ Description
Antibodies	
Alexa Fluor 488 Phalloidin	Thermo Fisher (#A12379). Used with 1:100 dilution (in which 1:400 recommended by the manufacture).
Rabbit anti-GFP polyclonal antibody	Medical & Biological Laboratories Co. Ltd. (#598). Used with 1: 500 dilution.
Goat anti-rabbit IgG Alexa 488	Thermo Fisher (#A-11008). Used with 1: 500 dilution.
Chemicals	
In-Fusion PCR cloning kit	Clontech (#639648)
KOD-Plus-Neo	Toyobo (#KOD-401)
10× Phosphate Buffered Saline	Nacalai tesque (#27575-31)
16% Formaldehyde	Pierce (#28906)
VECTASHIELD Mounting Medium	VECTOR LABORATORIES, INC. (#H-1000)
Plasmid	
pmScarlet_C1	Addgene (#85042). Used for template to amplify mScarlet sequence.
pUASTattB-LifeAct::GFP11x3	Used for backbone vector after excluding GFP11x3 sequence. Ref[1]
pUASTattB-LifeAct::mScarletx2	Expression vector of tandem mScarlet fused with actin-binding peptide, LifeAct. Generated in this study.
pUAST-LifeAct::EGFP	Expression vector of EGFP fused with actin-binding peptide, LifeAct. Ref [2]
Primers	sequence (5'-3')
SS#38	AGGGGGATCCACCGGTCGCCACCATGGTGAGCAAGGGCGAGGC
SS#39	GCCGCGGCCGCAGATCTACTTGTACAGCTCGTCCATGCCG
SS#40	CATGGTGGCGACCGGACCTCCGCCCTTGTACAGCTCGTCCATGCCG
Strain (*Drosophila melanogaster*)	
w[1118]	wild type control
UAS-lifeact::EGFP (III)	BDSC#57326. Expresses an actin-binding peptide (lifeact) fused to GFP under UAS control.
UAS-lifeact::EGFP(II)	Expresses lifeact fused to GFP under UAS control. Inserted at attP40 site of Chr 2. Generated in this study.
UAS-lifeact::mScarletx2 (II)	Expresses lifeact fused to tandem mScarlet under UAS control. Inserted at attP40 site of Chr 2. Generated in this study.
UAS-lifeact::mScarletx2 (III)	Expresses lifeact fused to tandem mScarlet under UAS control. Inserted at attP2 site of Chr 3. Generated in this study.
UASp-Utrophion::EGFP	Expresses an actin-binding peptide fused to GFP under UAS control. Gifted by Dr. Marta Llimargas. Ref [3]
UASp-RFP-RhoGEF2	Expresses RhoGEF2 fused to RFP under UAS control. Gifted by Dr. Jörg Grosshans. Ref [4]
UAS-GFP[S65T]	Expresses GFP[S65T] (used as a control) Ref [5]
UAS-RhoA-RNAi	BDSC#32383. RNAi for RhoA. Ref [6]
UAS-RhoA[N19]	BDSC #7328. Dominant negative form of RhoA.
UAS-zip[ROD]	Dominant negative form of zipper (Myosin II heavy chain). Ref [7]
Actn[14]/ FM7a	DGRC#107829. Null mutant for α-actnin
DAAM $^{MI04569\text{-}GFSTF.0}$	BDSC#60213. Endogenous DAAM is labeled by GFP. Ref [8]
Zasp52-GFP	BDSC#6838. Endogenous Zasp52 is labeled by GFP.
Actn-GFP	BDSC#51573. Endogenous α-actnin is labeled by GFP. Ref [9]
zip-GFP	BDSC#51564. Endogenous zip (Myosin II) is labeled by GFP. Ref [9]
Rok-mNeonGreen	Endogenous Rok is labeled by mNeonGreen. Gifted by Dr. Katja Roper. Ref [10]
Software and Algorithmes	
ImageJ-Fiji	LOCI (https://imagej.net/software/fiji/)
MATLAB 2022a	The MathWorks (https://mathworks.com)
OrientationJ	(http://bigwww.epfl.ch/demo/orientation/) Ref [11]
R	The R foundation (https://www.r-project.org/)
ZEN	Zeiss. Used for image acquisition and Airyscan processing.
Original ImageJ plugins	TPIV, AutoEnhance, CoordinateShift and CrossSectionViewer available at "https://signaling.riken.jp/tools/imagej-plugins/"

Supplementary Notes: Emergence of periodic circumferential actin cables from anisotropic fusion of actin nanoclusters during tubulogenesis

Sayaka Sekine, Mitsusuke Tarama, Housei Wada, Mustafa M Sami, Tatsuo Shibata, and Shigeo Hayashi
(Dated: December 12, 2023)

I. COARSE-GRAINED MODEL

We explain our coarse-grained molecular dynamics model of actin filaments, myosin motors, and α-actinin crosslinkers. The model is developed based on our previous model of actin filaments and myosin motors [12], which is in the same spirit as those in many other studies including those in Refs. [13, 14]. See also the references in Ref. [12] for other related studies of this type of modeling. We extend the model to include crosslinkers. Since the actin nanoclusters and stripes are observed in the vicinity of the apical membrane, we consider the actin cytoskeleton dynamics in a two-dimensional space along the membrane. We further assume a smooth membrane, and thus the membranes are not included explicitly.

We start by defining actin filaments, myosin motors, and α-actinin crosslinkers. First, actin filaments are polymer with polarity, and their diameter is about 10 nm and their persistent length is about 1 μm [15]. The length of the actin filaments in our experimental system is estimated about 100 nm (Supplement Figure 6a). Based on this estimation, we neglect the bending of actin filament and model a filament by a pair of two discrete particles connected by an elastic spring, which keeps the length of the filament (Fig. 4a in the main text).

The myosin II motor we consider is non-muscle myosin II filament, where a few tens of non-muscle myosin II forming a bipolar bundle with multiple heads on both ends [16]. The typical size of myosin II filaments is about 10 nm in width and 200 nm in length. We refer to the myosin II filament simply by motor hereafter. To represent the bipolar structure, we model a motor by a particle with two actin binding domains (ABD) (Fig. 4a in the main text). The two ABD can bind to two different filaments and walk actively along them.

α-actinin form an antiparallel dimer that crosslinks a pair of actin filaments like a motor, but they do not undergo active walking along the filaments unlike the motors [17]. The typical size of α-actinin dimer is about 6 nm in width and 36 nm in length. We model α-actinin dimer in the same way as the motor, namely by a particle with two actin binding domains (ABD) (Fig. 4a in the main text). The two ABD bind to a pair of actin filaments, but they do not walk along the actin filaments. To highlight the crosslinking function of the α-actinin dimers, we refer to them as crosslinkers hereafter.

The equations of motion for the filament, motor, and crosslinker particles are given by

$$\gamma^{\mathrm{f}} \frac{d\boldsymbol{r}^{\mathrm{f}}_{f,i}}{dt} = \boldsymbol{f}^{\mathrm{f,str}}_{f,i} + \boldsymbol{f}^{\mathrm{f,m}}_{f,i} + \boldsymbol{f}^{\mathrm{f,cl}}_{f,i} + \boldsymbol{\xi}^{\mathrm{f}}_{f,i}, \tag{S1}$$

$$\gamma^{\mathrm{m}} \frac{d\boldsymbol{r}^{\mathrm{m}}_{m}}{dt} = \sum_{h=0,1} \big(-\boldsymbol{f}^{\mathrm{m,str}}_{m,h} - \boldsymbol{f}^{\mathrm{m,bend}}_{m,h} \big) + \boldsymbol{\xi}^{\mathrm{m}}_{m}, \tag{S2}$$

$$\gamma^{\mathrm{cl}} \frac{d\boldsymbol{r}^{\mathrm{cl}}_{c}}{dt} = \sum_{h=0,1} \big(-\boldsymbol{f}^{\mathrm{cl,str}}_{c,h} - \boldsymbol{f}^{\mathrm{cl,bend}}_{c,h} \big) + \boldsymbol{\xi}^{\mathrm{cl}}_{c}. \tag{S3}$$

Here $\boldsymbol{r}^{\mathrm{f}}_{f,i}$ represents the position of the ith particle of filament f. Since the filament consists of two particles, $i = 1, 2$ and the particle $i = 0$ and 1 correspond to the minus and plus ends, respectively. $\boldsymbol{r}^{\mathrm{m}}_{m}$ is the particle position of motor m and $\boldsymbol{r}^{\mathrm{cl}}_{c}$ represents the particle position of crosslinker c. The coordinate $0 \le \alpha^{\mathrm{m}}_{m,h} \le 1$ along the filament f gives the position of the hth ABD ($h = 1, 2$) of motor m as $(1 - \alpha^{\mathrm{m}}_{m,h})\boldsymbol{r}^{\mathrm{f}}_{f,0} + \alpha^{\mathrm{m}}_{m,h}\boldsymbol{r}^{\mathrm{f}}_{f,1}$ (Fig. S1). Then, the equation of motion for the motor ABD reads

$$\zeta \ell^{\mathrm{f}}_{f} \frac{d\alpha^{\mathrm{m}}_{m,h}}{dt} = f^{\mathrm{walk}}, \tag{S4}$$

where $\ell^{\mathrm{f}}_{f} = |\boldsymbol{r}^{\mathrm{f}}_{f,1} - \boldsymbol{r}^{\mathrm{f}}_{f,0}|$ is the filament length. In the same way, the position of the hth ABD ($h = 1, 2$) of crosslinker c is specified by the coordinate $0 \le \alpha^{\mathrm{cl}}_{c,h} \le 1$ along the filament to which the crosslinker ABD binds. Note that the crosslinker ABD does not walk or slide along the filament to which it binds, and thus its equation of motion does not need to be considered. The filament index f is omitted hereafter for ease of notation.

Inertia terms are neglected because of the small size and velocity of the molecules; The typical sizes of the cytoskeletal filament, the motor, and the crosslinkers are less than submicrometers. The sliding velocity of the motor head is

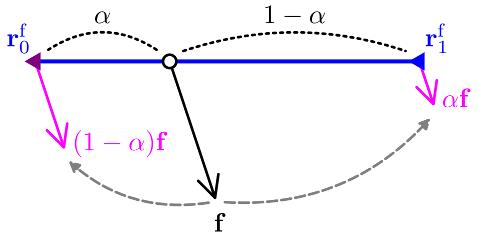

FIG. S1. Schematics explaining the position specified by the coordinate $0 \leq \alpha \leq 1$ along the filament, and the method to calculate the force $\boldsymbol{f}$ acting at the position α on the particles $\boldsymbol{r}_0^{\mathrm{f}}$ and $\boldsymbol{r}_1^{\mathrm{f}}$.

submicrometers per second. Note that the filament polarity is taken into account by the order of the filament particle indices, which gives the cue for the motor heads to walk actively towards the filament plus end. The filament, motor, and crosslinker particles experience friction and fluctuation from the surrounding cytosolic fluid, which satisfy the fluctuation-dissipation theorem; $\langle \boldsymbol{\xi}_i^{\mathrm{X}} \rangle = 0$ and $\langle \xi_{i,a}^{\mathrm{X}}(0)\xi_{j,b}^{\mathrm{X}}(t)\rangle = 2\gamma^{\mathrm{X}}\delta_{ij}\delta_{ab}\delta(t)$ with $\mathrm{X} = \{\mathrm{f}, \mathrm{m}, \mathrm{cl}\}$. Here δ_{ij} is the Kronecker delta that is 1 if $i = j$ and 0 if $i \neq j$. The cytosolic friction is defined using a cylindrical approximation [18] as $\gamma^{\mathrm{f}} = 3\pi\eta_{\mathrm{cyto}}(3d^{\mathrm{f}} + 2L_0^{\mathrm{f}}/5)$ and $\gamma^{\mathrm{m}} = 6\pi\eta_{\mathrm{cyto}}(3d^{\mathrm{m}} + L_0^{\mathrm{m}}/5)$ and $\gamma^{\mathrm{cl}} = 6\pi\eta_{\mathrm{cyto}}(3d^{\mathrm{cl}} + L_0^{\mathrm{cl}}/5)$, where η_{cyto} is the cytosolic viscosity and (d^{f}, L_0^{f}), (d^{m}, L_0^{m}), and (d^{cl}, L_0^{cl}) are the width and length of the filament, motor, and crosslinker, respectively. On the other hand, since the motor ABD are bound to and walk along filaments due to the motor walk force f^{walk}, they experience a sliding friction $\zeta\ell^{\mathrm{f}} d\alpha_{m,h}^{\mathrm{m}}/dt$.

The length of the filament is maintained by the stretching elasticity acting between the filament particles:

$$\boldsymbol{f}_i^{\mathrm{f,str}} = -\frac{\partial}{\partial \boldsymbol{r}_i^{\mathrm{f}}} U^{\mathrm{harm}}(\ell^{\mathrm{f}}; \kappa^{\mathrm{f,str}}, L_0^{\mathrm{f}}), \tag{S5}$$

where $U^{\mathrm{harm}}(r; \kappa, L_0) = (\kappa/2L_0)(r - L_0)^2$ is a harmonic potential with elastic modulus κ and rest length L_0.

Similarly, the stretching and bending forces act between the motor particles and motor ABD:

$$\boldsymbol{f}_{m,h}^{\mathrm{m,str}} = -\frac{\partial}{\partial \boldsymbol{r}_{m,h}^{\mathrm{m}}} \sum_h U^{\mathrm{harm}}(\ell_{m,h}^{\mathrm{m}}; \kappa^{\mathrm{m,str}}, L_0^{\mathrm{m}}/2), \tag{S6}$$

$$\boldsymbol{f}_{m,h}^{\mathrm{m,bend}} = -\frac{\partial}{\partial \boldsymbol{r}_{m,h}^{\mathrm{m}}} U^{\mathrm{bend}}(-\boldsymbol{\ell}_{m,0}^{\mathrm{m}}, \boldsymbol{\ell}_{m,1}^{\mathrm{m}}; k^{\mathrm{m,bend}}), \tag{S7}$$

where $\boldsymbol{\ell}_{m,h}^{\mathrm{m}} = \boldsymbol{r}_{m,h}^{\mathrm{m}} - \boldsymbol{r}_m^{\mathrm{m}}$. $U^{\mathrm{bend}}(\boldsymbol{r}_-, \boldsymbol{r}_+; k) = -k\hat{\boldsymbol{r}}_- \cdot \hat{\boldsymbol{r}}_+$ is bending energy with bending rigidity k. Here, we use the abbreviations $x = |\boldsymbol{x}|$ and $\hat{\boldsymbol{x}} = \boldsymbol{x}/|\boldsymbol{x}|$. Their contribution to the motor ABD, $\boldsymbol{f}_{m,h}^{\mathrm{m}} = \boldsymbol{f}_{m,h}^{\mathrm{m,str}} + \boldsymbol{f}_{m,h}^{\mathrm{m,bend}}$, is assigned to the filament particles with the geometric weight $\alpha_{m,h}^{\mathrm{m}}$, which is necessary because the binding position may be in between the filament particles (Fig. S1). Therefore, the force on the filament particle i from the motors reads $\boldsymbol{f}_i^{\mathrm{f,m}} = \delta_{i0}\sum_{m,h}(1 - \alpha_{m,h}^{\mathrm{m}})\boldsymbol{f}_{m,h}^{\mathrm{m}} - \delta_{i1}\sum_{m,h}\alpha_{m,h}^{\mathrm{m}}\boldsymbol{f}_{m,h}^{\mathrm{m}}$. Here the summations are calculated over the motor ABD bound to the filament.

Likewise, the length and the straightness of the crosslinkers are maintained by the stretching and bending force acting between the crosslinker particles and crosslinker ABD:

$$\boldsymbol{f}_{c,h}^{\mathrm{cl,str}} = -\frac{\partial}{\partial \boldsymbol{r}_{c,h}^{\mathrm{cl}}} \sum_h U^{\mathrm{harm}}(\ell_{c,h}^{\mathrm{cl}}; \kappa^{\mathrm{cl,str}}, L_0^{\mathrm{cl}}/2), \tag{S8}$$

$$\boldsymbol{f}_{c,h}^{\mathrm{cl,bend}} = -\frac{\partial}{\partial \boldsymbol{r}_{c,h}^{\mathrm{cl}}} U^{\mathrm{bend}}(-\boldsymbol{\ell}_{c,0}^{\mathrm{cl}}, \boldsymbol{\ell}_{c,1}^{\mathrm{cl}}; k^{\mathrm{cl,bend}}), \tag{S9}$$

where $\boldsymbol{\ell}_{c,h}^{\mathrm{cl}} = \boldsymbol{r}_{c,h}^{\mathrm{cl}} - \boldsymbol{r}_c^{\mathrm{cl}}$. Their contribution to the crosslinker ABD, $\boldsymbol{f}_{c,h}^{\mathrm{cl}} = \boldsymbol{f}_{c,h}^{\mathrm{cl,str}} + \boldsymbol{f}_{c,h}^{\mathrm{cl,bend}}$, is assigned to the filament particles with the geometric weight $\alpha_{c,h}^{\mathrm{cl}}$ as $\boldsymbol{f}_i^{\mathrm{f,cl}} = \delta_{i0}\sum_{c,h}(1 - \alpha_{c,h}^{\mathrm{cl}})\boldsymbol{f}_{c,h}^{\mathrm{cl}} - \delta_{i1}\sum_{c,h}\alpha_{c,h}^{\mathrm{cl}}\boldsymbol{f}_{c,h}^{\mathrm{cl}}$, where the summations are calculated over the crosslinker ABD bound to the filament.

These treatment of the forces from motors and crosslinkers acting on a filament ensure force and torque conservation of the filament. Note that the counter force of the motor sliding friction and motor walk force, which act on the filaments, vanishes because of the force balance equation (S4). Therefore, Eqs. (S1)–(S4) statistically satisfy the

force- and torque-free conditions, which are required for active systems including molecular motors [12] and migrating cells [19, 20]. See also Ref [12] for details as well as the derivation of Eq. (S4).

In addition to the equations of motion (S1)-(S4), we consider the following stochastic processes. The first one is the process of motor binding and unbinding. For simplicity, we assume that a motor takes either the bound state where both of the ABD are bound to filaments, or the free state where neither of the two ABD are bound to filaments. Unbinding of a motor ABD occurs in three cases. Firstly, a motor ABD that is bound to a filament unbinds stochastically at the rate of ω^{m}. Secondly, when a motor ABD reaches the end of a filament by actively walking along it, it unbinds instantaneously with the probability unity. Thirdly, a motor ABD unbinds with the probability unity when the other ABD of the motor unbinds. The free motors are assumed to diffuse sufficiently fast, so that they distribute uniformly. Then, a free motor binds to a randomly-selected pair of filaments whenever it find a position on each filament that are separated by the length of the motor L_0^{m}. Thus, this motor binding process alone causes no force on the system. The active force is generated in the cytoskeleton through the motor stretching and bending energies that are stored when motor ABD move along filaments.

Like the motors, we assume that a crosslinker takes either the bound state where both of the ABD are bound to filaments, or the free state where neither of the two ABD are bound to filaments. Unbinding of a crosslinker ABD that is bound to a filament unbinds stochastically at the rate of ω^{cl}, or with the probability unity when the other ABD of the crosslinker unbinds. The free crosslinkers are assumed to diffuse sufficiently fast, so that they distribute uniformly. Then, a free crosslinker binds to a randomly-selected pair of filaments whenever they find a position on each filament that are separated by the length of the crosslinker L_0^{cl}. Thus, like the motor binding process, this binding process of the crosslinkers alone causes no force on the system.

The third stochastic process is filament turnover that takes place at the rate of ω^{f}. The filament undergoing turnover is placed back into the system immediately at random position with random orientation, and all the motors and crosslinkers previously bound to it become free.

In order to include the effect of tube expansion, we introduce frictional asymmetry that has been proposed in the previous study using active gel model [21]. Since our experimental data suggests that the formin actin nucleator protein DAAM plays a key role to align the fused actin nanoclusters and actin stripes in the circumferential direction, we assume that the friction asymmetry affects only actin filaments. Then, Eq. (S1) is separated to the spatial components as

$$\gamma_x^{\mathrm{f}} \frac{dr_{f,i,x}^{\mathrm{f}}}{dt} = f_{f,i,x}^{\mathrm{f,str}} + f_{f,i,x}^{\mathrm{f,m}} + f_{f,i,x}^{\mathrm{f,cl}} + \xi_{f,i,x}^{\mathrm{f}}, \tag{S1a}$$

$$\gamma_y^{\mathrm{f}} \frac{dr_{f,i,y}^{\mathrm{f}}}{dt} = f_{f,i,y}^{\mathrm{f,str}} + f_{f,i,y}^{\mathrm{f,m}} + f_{f,i,y}^{\mathrm{f,cl}} + \xi_{f,i,y}^{\mathrm{f}}, \tag{S1b}$$

where $\gamma_y^{\mathrm{f}}/\gamma_x^{\mathrm{f}}$ measures the friction asymmetry. $\gamma_y^{\mathrm{f}}/\gamma_x^{\mathrm{f}} = 1$ corresponds to the symmetric case.

The set of time-evolution equations is solved numerically in the following manner; First, we calculate the turnover of the filaments, motors, and crosslinkers, and the position of the motor ABD is updated by solving Eq. (S4) with the Euler method. Then, Eqs. (S1)–(S3) are solved by using the fourth-order Runge-Kutta method. In both cases, the time increment is set to $dt = 5 \times 10^{-5}$. The simulation results are visualized by using OVITO Pro [22].

II. PARAMETER VALUES

The simulation parameters for the filaments, motors, and crosslinkers are set as summarized in Table S1, unless otherwise stated. Some of the parameters are compared with existing experimental measurements. We consider the dynamics in the membrane vicinity where the diffusion coefficient is about ten times smaller than that in bulk [26], and thus the effective cytosolic viscosity η_{cyto} in the membrane vicinity is assumed about 10 times larger than in the bulk. Therefore, we set $\eta_{\mathrm{cyto}} = 10^{-1}\,\mathrm{Pa\cdot sec}$ which is larger than the measured cytosolic viscosity 10^{-3}–10^{-1} $\mathrm{Pa\cdot sec}$ [27]. The simulation box is set as $3\,\mu\mathrm{m} \times 3\,\mu\mathrm{m}$ with periodic boundary conditions in both directions.

III. STEADY-STATE SOLUTIONS

We start the simulation from the initial condition where filaments are randomly placed with random orientation, to which motors and crosslinkers bind randomly following the binding rules. After relaxation that takes about 30-60 sec, we obtained steady-state solutions. By changing parameters including the number of motors and crosslinkers as well as the friction asymmetry, we found that the actin nanoclusters are formed under the existence of sufficient number of crosslinkers without friction asymmetry (Fig.4b). In contrast, actin stripes, which correspond to the regularly-spaced

TABLE S1. The summary of the simulation parameters. The ones indicated in bold font are the input parameters of the simulation.

	symbols		in silico	in vivo/in vitro	references
Cytosol					
**thermal energy**	k_BT		4.142×10^{-3} pN μm	at 300 K	
**cytosolic viscosity**	$\eta_{\rm cyto}$		1 Pa $\cdot$ sec		
Filament (actin)					
**number of filaments**	$N^{\rm f}$		4500	–	(estimated)
**diameter**	$d^{\rm f}$		0.01 μm	0.009 μm	[15]
**rest length**	$L_0^{\rm f}$		0.1 μm	$\approx$ 0.1 μm	(estimated)
**elastic modulus**	$\kappa^{\rm f,str}$		10 pN		
Young's modulus	$E^{\rm f,str}$	$= \kappa^{\rm f,str} / \big(\pi(\frac{1}{2}d^{\rm f})^2\big)$	$\sim 1.27 \times 10^5$ Pa	2×10^9 Pa	[23]
**turn over rate**	$\omega^{\rm f}$		0.1 sec^{-1}		
Motors (non-muscle myosin II filaments)					
**number of motors**	$N^{\rm m}$		900	–	
**diameter**	$d^{\rm m}$		0.01 μm	$\approx$ 0.01–0.03 μm	[16]
**rest length**	$L_0^{\rm m}$		0.2 μm	$\approx$ 0.2–0.3 μm	[16]
**stretching modulus**	$\kappa^{\rm m,str}$		10 pN		
Young's modulus	$E^{\rm m,str}$	$= \kappa^{\rm mot,str} / \big(\pi(\frac{1}{2}d^{\rm mot})^2\big)$	$\sim 1.27 \times 10^5$ Pa		
**bending rigidity**	$k^{\rm m,bend}$		0.1 pN μm		
persistence length	$\ell^{\rm m,bend}$	$= k^{\rm m,bend} \ell_0^{\rm m} / 2k_BT$	~ 2.4 μm		
**walk force**	$f^{\rm walk}$		0.05 pN	$\lessapprox$ a few pN (stall force)	[24]
**sliding friction**	ζ		0.1 pN sec /μm	$\lessapprox$ 0.1 pN sec /μm	[25]
**turn over rate**	$\omega^{\rm m}$		0.01 sec^{-1}		
Crosslinkers (α-actinin)					
**number of crosslinkers**	$N^{\rm cl}$		6750	–	
**diameter**	$d^{\rm cl}$		0.01 μm	$\approx$ 0.006 μm	[17]
**rest length**	$L_0^{\rm cl}$		0.035 μm	0.036 μm	[17]
**stretching modulus**	$\kappa^{\rm cl,str}$		10 pN		
Young's modulus	$E^{\rm cl,str}$	$= \kappa^{\rm cl,str} / \big(\pi(\frac{1}{2}d^{\rm cl})^2\big)$	$\sim 1.27 \times 10^5$ Pa		
**bending rigidity**	$k^{\rm cl,bend}$		0.57 pN μm		
persistence length	$\ell^{\rm cl,bend}$	$= k^{\rm cl,bend} \ell_0^{\rm cl} / 2k_BT$	~ 24.1 μm		
**turn over rate**	$\omega^{\rm cl}$		0.01 sec^{-1}		

actin cables found in the experiment, are obtained under the existence of friction asymmetry and sufficient number of motors. See also Supplementary Movie 4. We discuss how these structures are organized in more detail in this section.

III.1. Actin nanoclusters

First, we consider the simplest case with filaments and crosslinkers in the absence of friction asymmetry, in which the actin nanoclusters are formed. One key component for the actin nanocluster formation is the crosslinkers. In fact, if the number of crosslinkers is sufficiently large ($N^{\rm cl} = 6750$), the filaments organize into clusters as shown in Supplement Figure 7a1. See also the snapshots in Fig. 4b in the main text and Supplementary Movie 4. The size of these clusters is about 200-300 nm, which is comparable to the actin nanoclusters observed in the experiment (Fig. 4c in the main text and Supplement Figure 6b). If the number of crosslinkers is small, however, the filaments do not form nanoclusters but are distributed randomly.

This brings us to a question whether these nanoclusters are also formed under the existence of motors. To check this point, we perform a simulation with a finite number of motors (Supplement Figure 7a2). When the number of motors is small ($N^{\rm m} = 100$), the nanoclusters are still observed. For a large number of motors ($N^{\rm m} = 900$), however, the filaments form a labyrinth pattern. From these, we conclude that the motors are not necessary for the nanocluster formation. A small number of motors do not alter the structure, while too many of them change the pattern to a

labyrinth.

To further investigate which factor is important for the cluster formation, we focus on the case without motors and vary the length and turnover rate of the crosslinkers as well as those of the filaments. If the turnover rate of the crosslinkers is low ($\omega^{\mathrm{cl}} = 0.001$ and 0.01), the filaments organized into nanoclusters (Supplement Figure 7a3). In contrast, for a high turnover rate of the crosslinkers ($\omega^{\mathrm{cl}} = 0.03$), no clear clusters are observed and the filaments are distributed randomly. These results indicate that the crosslinkers play an important role in the clustering of filaments. That is, the crosslinkers connect the filaments and tend to keep them close to each other, leading to the effective attraction of filaments. However, this effective attraction of filaments reduces as the crosslinker turnover rate increases, resulting in the random distribution of filaments.

In the case of a rapid filament turnover ($\omega^{\mathrm{f}} = 0.5$), the filament distribution becomes rather uniform (Supplement Figure 7a4). As the filament turnover rate decreases, however, nanoclusters appear ($\omega^{\mathrm{f}} = 0.1$). Further decreasing ω^{f} makes the nanocluster structure more compact and the number of the clusters decreases, leaving a clear larger space void of filaments between clusters ($\omega^{\mathrm{f}} = 0.01$). See also Supplement Figure 6c and Supplementary Table 7. These results suggest that the filament turnover enhances the effective filament diffusion, which acts against the effective attraction to control the distance between the clusters.

We next consider the role of the length of the filaments and crosslinkers. On the one hand, as the crosslinker length increases, the clusters become loose (Supplement Figure 7a5 $L_0^{\mathrm{cl}} = 0.04$), and eventually no clear clusters are observed ($L_0^{\mathrm{cl}} = 0.06$). This suggests that the short length of the crosslinkers is essential to keep the filaments close enough to each other. On the other hand, if the filament length is decreased, we still observe clustering of the filaments (Supplement Figure 7a6 $L_0^{\mathrm{f}} = 0.075$). This result seems counterintuitive as the ratio of the crosslinker length with respect to the filament length becomes large in the case of short filaments. Since the number of filaments is kept constant, however, the total length of the filaments, i.e. the total amount of actin in the system also decreases with the filament length. This is the reason why the clusters are still observed in the case of short filaments (Supplement Figure 7a6 $L_0^{\mathrm{f}} = 0.075$). This also explains the result of the long filaments, where the clusters start to connect to each other ($L_0^{\mathrm{f}} = 0.125$).

In summary, filaments and a sufficient number of crosslinkers self-organize into nanoclusters in the absence or under the existence of a small number of motors. The results suggest that the clusters are caused by the competition between the effective attraction of the filaments due to the crosslinkers and the effective diffusion of the filaments. The sufficient effective attraction between the filaments connected by crosslinkers enables the filaments being further connected by other crosslinkers if there are more filaments and free crosslinkers in the neighbour. Therefore, a small number of crosslinkers, or high turnover rate of the crosslinkers, both of which decrease the effective attraction, result in a random or uniform filament distribution without any clear pattern. The effective filament diffusion controls the distance between the clusters. A high filament turnover rate enhances the effective diffusion, which disperses the filaments, leaving no cluster pattern. The length of the crosslinkers and the filament density are also important to make the clusters compact and well separated from each other. Finally, a small number of motors does not strongly affect the cluster pattern, although too many of them changes the structure to a labyrinth pattern.

III.2. Actin stripes

Here, we focus on the formation of the stripe pattern.

We start from the labyrinth pattern that appears from the filaments mixed with sufficient number of crosslinkers and motors (Supplement Figure 7a2 $N^{\mathrm{m}} = 900$). The labyrinth pattern is a connected structure consisting of actin filaments, crosslinkers and motors without any specific direction, whereas the stripe pattern is extended in the circumferential direction. To provide the external cue, we include friction asymmetry that affects only the filaments following the previous study [21]. The results explained in the previous section III.1 correspond to the case where the friction asymmetry $\gamma_y^{\mathrm{f}}/\gamma_x^{\mathrm{f}}$ is unity. As the friction asymmetry $\gamma_y^{\mathrm{f}}/\gamma_x^{\mathrm{f}}$ increases from unity, the connected structure of filaments tends to align in the direction of higher friction (Supplement Figure 7b1). For $\gamma_y^{\mathrm{f}}/\gamma_x^{\mathrm{f}} \gtrsim 1.5$, we obtained a stripe pattern.

Next, we study the effect of the motors on the stripe pattern formation under the existence of friction asymmetry. Starting from the stripe pattern, we first decrease the number of motors. Then, the connection of the pattern gets worse (Supplement Figure 7b2 $N^{\mathrm{m}} = 300$). By further decreasing the number of motors, the stripe pattern eventually disappears, leaving many discrete clusters that are slightly elongated in the higher friction direction ($N^{\mathrm{m}} = 100$ and 0). See also Figs. 4b (2-M) and 4c and Supplementary Movie 4.

To make more clear the role of the motors, we change the motor walk force. When the motor walk force is absent, the filaments exhibit a connected structure without clear directionality, i.e. a labyrinth pattern (Supplement Figure 7b3 $f^{\mathrm{walk}} = 0$). As the motor activity increases, the structure aligns in the higher friction direction, leading to a stripe pattern ($f^{\mathrm{walk}} = 0.001$ and 0.005). Further increasing motor walk force, the structure become loose and

no clear pattern is observed ($f^{\mathrm{walk}} = 0.05$). This is probably because the higher motor walk force adds more energy to the system and enhances the effective diffusion of the filaments, which makes it more difficult to maintain the self-organized structure.

These results suggest that, in the absence of motors or under the existence of small number of motors, the filaments and crosslinkers organize into nanoclusters, which react to the friction asymmetry and tend to elongate in the higher friction direction. Then, as the number of motors increases, these nanoclusters are connected by motors and eventually the pattern becomes stripes aligned in the higher friction direction. This leads to a question whether the stripe pattern appears in the presence of motors and friction asymmetry for the parameter region where the filaments fail to form clusters in the absence of the motors and friction asymmetry. To answer this question, we vary the turnover rate and the length of filaments and crosslinkers, which influence the cluster formation in the absence of motors.

First, we change the turnover rate of crosslinkers and filaments starting from the stripe pattern for $\omega^{\mathrm{cl}} = 0.01$ and $\omega^{\mathrm{f}} = 0.1$. (Supplement Figure 7b4 and 7b5). On the one hand, for a high turnover rate of crosslinkers or filaments, where no clusters are observed in the absence of friction asymmetry, filaments distribute randomly (Supplement Figure 7b4 $\omega^{\mathrm{cl}} = 0.02$ and Supplement Figure 7b5 $\omega^{\mathrm{f}} = 0.2$). On the other hand, by decreasing ω^{cl}, the stripe pattern becomes more compact in width ($\omega^{\mathrm{cl}} = 0.005$), and by further decreasing ω^{cl}, the connection of the pattern gets worse ($\omega^{\mathrm{cl}} = 0.001$). For a lower filament turnover rate, the filaments exhibit a compact stripe pattern ($\omega^{\mathrm{f}} = 0.05$). For a very low turnover rate ($\omega^{\mathrm{f}} = 0.01$), the filaments form clusters.

Next, we consider the impact of the length of filaments and crosslinkers on the stripe pattern formation. When the length of crosslinkers or filaments are increased, the stripe structure becomes loose (Supplement Figure 7b6 and 7b7). For a shorter filament length, however, the stripe structure gets more compact (Supplement Figure 7b6 $L_0^{\mathrm{f}} = 0.075$). For very short filaments, many small clusters are formed that are connected by motors (Supplement Figure 7b7 $L_0^{\mathrm{f}} = 0.05$). This is probably due to the decrease in filament density, i.e. the decrease in the total amount of actin molecules. In contrast, for a shorter crosslinker length, the stripe structure again becomes loose (Supplement Figure 7b6 $L_0^{\mathrm{cl}} = 0.03$). This may be because the crosslinkers are now too short to connect filaments.

To summarize, the actin nanoclusters, that appear as a result of the competition between the effective diffusion and the effective attraction acting between the filaments due to crosslinkers, react to the friction asymmetry and tend to elongate in the higher friction direction. Then, the motors connect these elongated actin nanoclusters and form stripes that align in the direction of higher friction. Therefore, the motors and the friction asymmetry are essential for the transition from the clusters to the stripe pattern. In fact, in the absence of the friction asymmetry but in the presence of motors, the structure loses preferred direction and the labyrinth pattern appears. In the absence or under the existence of small number of motors, the filaments form discrete clusters. In addition, the balance between the effective attraction of filaments due to crosslinkers and their effective diffusion is important for the stripe formation. On the one hand, on the conditions where the filaments fail to form clusters in the absence of motors and friction asymmetry, the stripe pattern is not obtained even in the presence of motors and friction asymmetry. On the other hand, for the parameters where the filaments form very compact clusters in the absence of motors and friction asymmetry, the clusters still remain in the presence of motors and friction asymmetry, and the transition to the stripe pattern does not occur.

Supplementary References

[1] Kamiyama, Sekine et al., Versatile protein tagging in cells with split fluorescent protein, *Nat Commun*, **7**, 11046 (2016)

[2] Dong et al., Rab9 and retromer regulate retrograde trafficking of luminal protein required for epithelial tube length control, *Nat Commun,* **4**, 1358 (2013)

[3] Rauzi et al., Planar polarized actomyosin contractile flows control epithelial junction remodelling, *Nature*, **468** (7237): 1110-4 (2010)

[4] Wenzl et al., Localization of RhoGEF2 during Drosophila cellularization is developmentally controlled by Slam, *Mech Dev.*, **127** (7-8): 371-84 (2010)

[5] Hayashi et al., GETDB, a database compiling expression patterns and molecular locations of a collection of Gal4 enhancer traps, *Genesis*, **34** (1-2): 58-61 (2002)

[6] Perkins et al., The Transgenic RNAi Project at Harvard Medical School: Resources and Validation, *Genetics*, **201** (3): 843-52 (2015)

[7] Franke et al., Nonmuscle myosin II is required for cell proliferation, cell sheet adhesion and wing hair morphology during wing morphogenesis, *Dev Biol*, **345** (2): 117-32 (2010)

[8] Nagarkar-Jaiswal et al., A library of MiMICs allows tagging of genes and reversible, spatial and temporal knockdown of proteins in Drosophila, *eLife*, **31**:4:e05338 (2015)

[9] Buszczak et al., The carnegie protein trap library: a versatile tool for Drosophila developmental studies, *Genetics*, **175** (3): 1505-31 (2006)

[10] Sidor et al., Rho-Kinase Planar Polarization at Tissue Boundaries Depends on Phospho-regulation of Membrane Residence Time, *Dev Cell*, **10**;52(3): 365-378.e7. (2020)

[11] Püspöki et al., Transforms and Operators for Directional Bioimage Analysis: A Survey, *Adv. Anat. Embryol. Cell Biol.*, **219**:69-93 (2016)

[12] M. Tarama and T. Shibata, Pattern formation and the mechanics of a motor-driven filamentous system confined by rigid membranes, Phys. Rev. Research **4**, 043071 (2022).

[13] F. Ziebert and I. S. Aranson, Rheological and structural properties of dilute active filament solutions, *Phys. Rev.* E **77**, 011918 (2008).

[14] J. A. Åström, P. B. S. Kumar, I. Vattulainen, and M. Karttunen, Strain hardening,

avalanches, and strain softening in dense cross-linked actin networks, *Phys. Rev.* E **77**, 051913 (2008).

[15] Q. Wen and P. A. Janmey, Polymer physics of the cytoskeleton, *Current Opinion in Solid State and Materials Science* **15**, 177 (2011).

[16] C. G. Vasquez, S. M. Heissler, N. Billington, J. R. Sellers, and A. C. Martin, *Drosophila* non-muscle myosin ii motor activity determines the rate of tissue folding, *eLife* **5**, e20828 (2016).

[17] E. Ribeiro, N. Pinotsis, A. Ghisleni, A. Salmazo, P. Konarev, J. Kostan, B. Sjöblom, C. Schreiner, A. Polyansky, E. Gk- ougkoulia, M. Holt, F. Aachmann, B. Žagrović, E. Bordignon, K. Pirker, D. Svergun, M. Gautel, and K. Djinović-Carugo, The structure and regulation of human muscle α-actinin, *Cell* **159**, 1447 (2014).

[18] T. Kim, W. Hwang, H. Lee, and R. D. Kamm, Computational analysis of viscoelastic properties of crosslinked actin networks, *PLOS Computational Biology* **5**, 1 (2009).

[19] M. Tarama and R. Yamamoto, Mechanics of cell crawling by means of force-free cyclic motion, *Journal of the Physical Society of Japan* **87**, 044803 (2018), https://doi.org/10.7566/JPSJ.87.044803.

[20] M. Tarama, K. Mori, and R. Yamamoto, Mechanochemical subcellular-element model of crawling cells, *Frontiers in Cell and Developmental Biology* **10**, 10.3389/fcell.2022.1046053 (2022).

[21] E. Hannezo, B. Dong, P. Recho, J.-F. Joanny, and S. Hayashi, Cortical instability drives periodic supracellular actin pattern formation in epithelial tubes, *Proceedings of the National Academy of Sciences* **112**, 8620 (2015), https://www.pnas.org/doi/pdf/10.1073/pnas.1504762112.

[22] A. Stukowski, Visualization and analysis of atomistic simulation data with ovito–the open visualization tool, *Modelling and Simulation in Materials Science and Engineering* **18**, 015012 (2009).

[23] H. Kojima, A. Ishijima, and T. Yanagida, Direct measurement of stiffness of single actin filaments with and with- out tropomyosin by in vitro nanomanipulation, *Proceedings of the National Academy of Sciences* **91**, 12962 (1994), https://www.pnas.org/content/91/26/12962.full.pdf.

[24] S. Chaen, J. Inoue, and H. Sugi, The force-velocity relationship of the atp-dependent actin-myosin sliding causing cyto- plasmic streaming in algal cells, studied using a centrifuge microscope., *Journal of Experimental Biology* **198**, 1021 (1995),https://journals.biologists.com/jeb/article-pdf/198/4/1021/1231870/1021.pdf.

[25] K. Tawada and K. Sekimoto, A physical model of atp-induced actin-myosin movement in vitro, *Biophysical Journal* **59**, 343 (1991).

[26] U. Golebiewska, M. Nyako, W. Woturski, I. Zaitseva, S. McLaughlin, and T. U.

Martin, Diffusion coefficient of fluorescent phosphatidylinositol 4,5-bisphosphate in the plasma membrane of cells, *Molecular Biology of the Cell* **19**, 1663 (2008), https://doi.org/10.1091/mbc.e07-12-1208.

[27] P. Valberg and H. Feldman, Magnetic particle motions within living cells. measurement of cytoplasmic viscosity and motile activity, *Biophysical Journal* **52**, 551 (1987).
