# Supplementary material for: Emergence of periodic circumferential actin cables from the anisotropic fusion of actin nanoclusters during tubulogenesis
Source: Nat Commun. 2024 Jan 24;15:464. doi: 10.1038/s41467-023-44684-z (PMC10808230; doi:10.1038/s41467-023-44684-z)
Supplement: Supplementary file 3 — Description of Additional Supplementary Files [file 41467_2023_44684_MOESM3_ESM.pdf]

Description of Additional Supplementary Files

File name: Supplementary Data 1
Description: List of RNAi strains for the 1st screen (crossed with btl-Gal4).

File name: Supplementary Data 2
Description: List of protein complexes identified in the protein network constructed by MIST.

File name: Supplementary Movie 1
Description: Actin cable formation in tracheal cells. Confocal time-lapse images showing the actin pattern change in the apical cortex of tracheal cells in stage 15 *Drosophila* embryo. 0.32 s/frame for 50 frames (15 s) at -40, -30, -20, -10, and 0 min of actin cable formation. Genotype is *btl>lifeact::GFP*. Scale bar, 2 j.tm.

File name: Supplementary Movie 2
Description: Actin dynamics in tracheal cells (-40 min). The fluorescent and binarized time-lapse images in square ROI (2.16 j.tm per side) at -40 min, when actin nanoclusters exhibit isotropic motion. Genotype is *btl>lifeact::GFP*. Scale bar, 0.5 j.tm.

File name: Supplementary Movie 3
Description: Actin dynamics in tracheal cells (0 min). The fluorescent and binarized time-lapse images in square ROI (2.16 j.tm per side) at 0 min, when the actin nanoclusters exhibit anisotropic motion and formation of periodic cables. Genotype is *btl>lifeact::GFP*. Scale bar, 0.5 j.tm.

File name: Supplementary Movie 4
Description: Animation of the simulation results showing the self-organization of actin nanoclusters. The actin filaments are dispersed with a low number of crosslinkers (1-L), however, with sufficient numbers of crosslinkers, the regularly spaced nanoclusters are self-organized (1-M). The impacts of anisotropic friction (2-M), motors (3-M), or both (4-M) on the

cluster (1-M) are also shown. The blue line indicates actin filament and the red line indicates myosin motors. The conditions correspond to the cases in Fig. 4b.
