# Supplementary material for: Emergence of periodic circumferential actin cables from the anisotropic fusion of actin nanoclusters during tubulogenesis
Source: Nat Commun. 2024 Jan 24;15:464. doi: 10.1038/s41467-023-44684-z (PMC10808230; doi:10.1038/s41467-023-44684-z)
Supplement: Supplementary file 10 — Reporting Summary [file 41467_2023_44684_MOESM10_ESM.pdf]

nature portfolio

Corresponding author(s): Sayaka Sekine, Mitsusuke Tarama

Last updated by author(s): Dec 13, 2023

Reporting Summary

Nature Portfolio wishes to improve the reproducibility of the work that we publish. This form provides structure for consistency and transparency in reporting. For further information on Nature Portfolio policies, see our Editorial Policies and the Editorial Policy Checklist.

Statistics

For all statistical analyses, confirm that the following items are present in the figure legend, table legend, main text, or Methods section.

n/a	Confirmed	
☐	☒	The exact sample size (n) for each experimental group/condition, given as a discrete number and unit of measurement
☐	☒	A statement on whether measurements were taken from distinct samples or whether the same sample was measured repeatedly
☐	☒	The statistical test(s) used AND whether they are one- or two-sided<br>*Only common tests should be described solely by name; describe more complex techniques in the Methods section.*
☒	☐	A description of all covariates tested
☐	☒	A description of any assumptions or corrections, such as tests of normality and adjustment for multiple comparisons
☐	☒	A full description of the statistical parameters including central tendency (e.g. means) or other basic estimates (e.g. regression coefficient) AND variation (e.g. standard deviation) or associated estimates of uncertainty (e.g. confidence intervals)
☐	☒	For null hypothesis testing, the test statistic (e.g. F, t, r) with confidence intervals, effect sizes, degrees of freedom and P value noted<br>*Give P values as exact values whenever suitable.*
☒	☐	For Bayesian analysis, information on the choice of priors and Markov chain Monte Carlo settings
☒	☐	For hierarchical and complex designs, identification of the appropriate level for tests and full reporting of outcomes
☐	☒	Estimates of effect sizes (e.g. Cohen's d, Pearson's r), indicating how they were calculated

Our web collection on statistics for biologists contains articles on many of the points above.

Software and code

Policy information about availability of computer code

Data collection	ZEN software (Zeiss Microscopy). Custom code for theoretical analysis is available upon request. A detailed description is provided in the Supplementary Notes.
Data analysis	ImageJ Fiji, an ImageJ plugin OrientationJ, R, Matlab, MIST (ver 5.0, https://fgrtools.hms.harvard.edu/MIST/), and homemade ImageJ plugins (ZT interpolation, TPIV, CorssSectionViewer) that are available at https://signaling.riken.jp/tools/imagej-plugins/.

For manuscripts utilizing custom algorithms or software that are central to the research but not yet described in published literature, software must be made available to editors and reviewers. We strongly encourage code deposition in a community repository (e.g. GitHub). See the Nature Portfolio guidelines for submitting code & software for further information.

Data

Policy information about availability of data

All manuscripts must include a data availability statement. This statement should provide the following information, where applicable:

- Accession codes, unique identifiers, or web links for publicly available datasets
- A description of any restrictions on data availability
- For clinical datasets or third party data, please ensure that the statement adheres to our policy

All data will be shared in https://doi.org/10.24631/ssbd.repos.2023.12.330. They are also available from the corresponding author upon request.

Research involving human participants, their data, or biological material

Policy information about studies with human participants or human data. See also policy information about sex, gender (identity/presentation), and sexual orientation and race, ethnicity and racism.

Reporting on sex and gender	N/A
Reporting on race, ethnicity, or other socially relevant groupings	N/A
Population characteristics	N/A
Recruitment	N/A
Ethics oversight	N/A

Note that full information on the approval of the study protocol must also be provided in the manuscript.

Field-specific reporting

Please select the one below that is the best fit for your research. If you are not sure, read the appropriate sections before making your selection.

☒ Life sciences ☐ Behavioural & social sciences ☐ Ecological, evolutionary & environmental sciences

For a reference copy of the document with all sections, see nature.com/documents/nr-reporting-summary-flat.pdf

Life sciences study design

All studies must disclose on these points even when the disclosure is negative.

Sample size	No sample-size calculation was performed. Sample sizes were chosen based on our previous studies (Ogura et al, Dev Cell, 2018; Hannezo et al., PNAS, 2015) and those are similar to other studies in this research field.
Data exclusions	Image data were excluded if the cells showed quite weak signals due to failure in sample preparation.
Replication	At least three animals were examined to collect data points and to ensure the reproducibility in each experiment. All attempts at replication were successful.
Randomization	Samples were grouped by genotype.
Blinding	No blinding was performed since it is impossible to retrieve the samples mounted on the glass bottom dish after live imaging.

Reporting for specific materials, systems and methods

We require information from authors about some types of materials, experimental systems and methods used in many studies. Here, indicate whether each material, system or method listed is relevant to your study. If you are not sure if a list item applies to your research, read the appropriate section before selecting a response.

Materials & experimental systems

n/a	Involved in the study	
☐	☒	Antibodies
☒	☐	Eukaryotic cell lines
☒	☐	Palaeontology and archaeology
☐	☒	Animals and other organisms
☒	☐	Clinical data
☒	☐	Dual use research of concern
☒	☐	Plants

Methods

n/a	Involved in the study	
☒	☐	ChIP-seq
☒	☐	Flow cytometry
☒	☐	MRI-based neuroimaging

Antibodies

Antibodies used	Alexa Fluor 488 Phalloidin (ThermoFisher, A12379), rabbit anti-GFP polyclonal antibody (Medical & Biological Laboratories Co., Ltd, #598), and Goat anti-rabbit IgG Alexa488 (Thermo Fisher, A-11008).

Validation	Alexa Fluor 488 Phalloidin is widely used and cited in more than 189 papers, that are introduced in the manufacture' page (https://www.thermofisher.com/order/catalog/product/jp/ja/A12379). The rabbit anti-GFP polyclonal antibody is also widely used antibody (https://www.mblintl.com/products/598/). The Goat anti-rabbit IgG Alexa488 antibody is also widely used antibody (https://www.thermofisher.com/antibody/product/Goat-anti-Rabbit-IgG-H-L-Cross-Adsorbed-Secondary-Antibody-Polyclonal/A-11008).

Animals and other research organisms

Policy information about studies involving animals; ARRIVE guidelines recommended for reporting animal research, and Sex and Gender in Research

Laboratory animals	Drosophila melanogaster of embryonic stages 14 to 17 (about 12 to 18 hours after egg laying) was used in this study. All strains are listed in the Supplementary Table 6. The age of female virgin flies used for RNAi screen crosses was between 1 to 7 days after eclosion. The age of male flies was not specified.
Wild animals	This study did not involve wild animals.
Reporting on sex	This information has not been collected.
Field-collected samples	This study did not involve samples collected from field.
Ethics oversight	RIKEN BDR

Note that full information on the approval of the study protocol must also be provided in the manuscript.
